# Urine-derived cells provide a readily accessible cell type for feeder-free mRNA reprogramming

**DOI:** 10.1038/s41598-018-32645-2

**Published:** 2018-09-25

**Authors:** A. Gaignerie, N. Lefort, M. Rousselle, V. Forest-Choquet, L. Flippe, V. Francois–Campion, A. Girardeau, A. Caillaud, C. Chariau, Q. Francheteau, A. Derevier, F. Chaubron, S. Knöbel, N. Gaborit, K. Si-Tayeb, L. David

**Affiliations:** 1 SFR-SANTE, iPSC core facility, INSERM, CNRS, UNIV Nantes, CHU Nantes, Nantes, France; 2 IPS Platform, Institut Imagine, INSERM, Paris Descartes-Sorbonne University, Paris Cité University, Paris, France; 3 Institut du thorax, INSERM, CNRS, UNIV Nantes, Nantes, France; 4 CRTI, INSERM, Université de Nantes, Nantes, France; 5 ITUN, CHU Nantes, Nantes, France; 6 LabEx IGO “Immunotherapy, Graft, Oncology”, Nantes, France; 7 Institut Clinident, Bat Laennec, Domaine du petit arbois, 13592 Aix en Provence Cedex 3, France; 8 Miltenyi Biotec GmbH, 51429 Bergisch Gladbach, Germany

## Abstract

Over a decade after their discovery, induced pluripotent stem cells (iPSCs) have become a major biological model. The iPSC technology allows generation of pluripotent stem cells from somatic cells bearing any genomic background. The challenge ahead of us is to translate human iPSCs (hiPSCs) protocols into clinical treatment. To do so, we need to improve the quality of hiPSCs produced. In this study we report the reprogramming of multiple patient urine-derived cell lines with mRNA reprogramming, which, to date, is one of the fastest and most faithful reprogramming method. We show that mRNA reprogramming efficiently generates hiPSCs from urine-derived cells. Moreover, we were able to generate feeder-free bulk hiPSCs lines that did not display genomic abnormalities. Altogether, this reprogramming method will contribute to accelerating the translation of hiPSCs to therapeutic applications.

## Introduction

Human pluripotent stem cells (hPSCs), either embryonic stem cells (hESCs) derived from human blastocysts, or induced pluripotent stem cells (hiPSCs) derived from somatic cells, have the ability to differentiate into all cell types comprising an adult organism, thus offering a great promise for regenerative medicine. Clinical trials are rapidly moving forward with human embryonic stem cells (hESC), paving the way for future clinical trials using hiPSCs^1^. Nonetheless, realizing the promises of hiPSCs in regenerative medicine rests on overcoming several hurdles, such as the genomic instability in hiPSCs, the variability in differentiation potential among hiPSCs and the tolerance of the immune system for auto- or allo-graft of cells differentiated from hiPSCs^2^. These hurdles are partly linked to the reprogramming system employed and the parental cell lines used to generate iPSCs.

Since their discovery, hiPSCs have been extensively compared to their embryonic-derived counterparts (hESCs). Both cell types are functionally pluripotent. However, reports from 2007 to 2012 shed light on molecular discrepancies between hESCs and hiPSCs. An overarching conclusion from these studies is that both hiPSC lines and hESC lines could highly vary in quality, thus making the selection of “good clones” a major challenge^3^. In other words, there is a need to limit the false-positive clones, which would not be used because of genomic abnormalities. To address this, an array of non-insertional reprogramming methods has been developed to replace the traditional retro- or lentivirus-based reprogramming protocols^4^. Since then, numerous studies have reported deriving iPS cells by delivering the reprogramming factors, Oct4, Klf4, Myc and Sox2, to somatic cells by mRNA, episome, Sendai virus or purified proteins^5–8^. The choice of one reprogramming method over another is based on the efficiency of the method in the parental cell source, preservation of the genomic integrity in the cell types during reprogramming as well as other potential caveats. Schlaeger and colleagues assessed these factors and demonstrated that mRNA reprogramming is the most efficient method based on the number of iPS clones obtained per cells seeded^9^. They have also shown that mRNA reprogramming is the method with the least impact on genome stability. Despite these apparent advantages, only about 60% of patient skin fibroblast specimens were reported to be amenable to mRNA reprogramming.

Reprogramming of human fibroblasts with mRNA was first achieved in 2010^5–10^. The original protocol required daily transfections of the reprogramming factors for 20 days. This protocol was subsequently improved, requiring less than 12 transfections, and allowing feeder-free derivation of hiPSCs^11–13^, thus reducing the complexity of the protocol and paving the way for GMP production of hiPSCs. To date, the main source of cells for mRNA reprogramming is skin fibroblasts, a cell type that tolerates genomic rearrangements, that will be present in the fibroblasts and therefore in the subsequent hiPSC lines^2^. However, sourcing skin fibroblasts requires medical intervention and aftercare. This is a drawback in cases where the donor is a healthy child, control to a diseased relative, or when repeated biopsies might be required in order to generate hiPSCs with specific immunological features. Thus, there is a need to develop an efficient reprogramming method for a more easily available cell source such as peripheral blood mononuclear cells (PBMCs), which can be obtained through less invasive means.

In this study, we explored alternative sources of starting cell types for mRNA reprogramming. Among adherent cell types that could be easily and non-invasively collected at cell banks, we identified dental pulp cells, which are collected following wisdom teeth removal, and urine-derived cells^14^. We successfully generated hiPSCs in feeder or feeder-free conditions from both cell types. The results prompted us to evaluate bulk reprogramming, i.e., generation of hiPSCs lines from multiple clones. The advantages of bulk reprogramming are that it is less labour-intensive and limits negative clonal effects occurring during reprogramming^15^, while the drawback being the increased risk of having varying genomic abnormalities in subclonal populations of a heterogenous hiPSC cell line. Single-nucleotide polymorphism (SNP) analysis revealed that bulk hiPSCs from urine-derived cells did not present genomic duplication or deletion. Whereas, Sendai reprogramming of fibroblasts and PBMCs yielded 18% copy number variation (CNV) rate. Our work will extend the versatility of mRNA reprogramming method and help clear the remaining roadblocks to the therapeutic application of hiPSCs.

## Results

### Comparing mRNA reprogramming of urine-derived cells, dental pulp cells and fibroblasts

**Figure 1:**
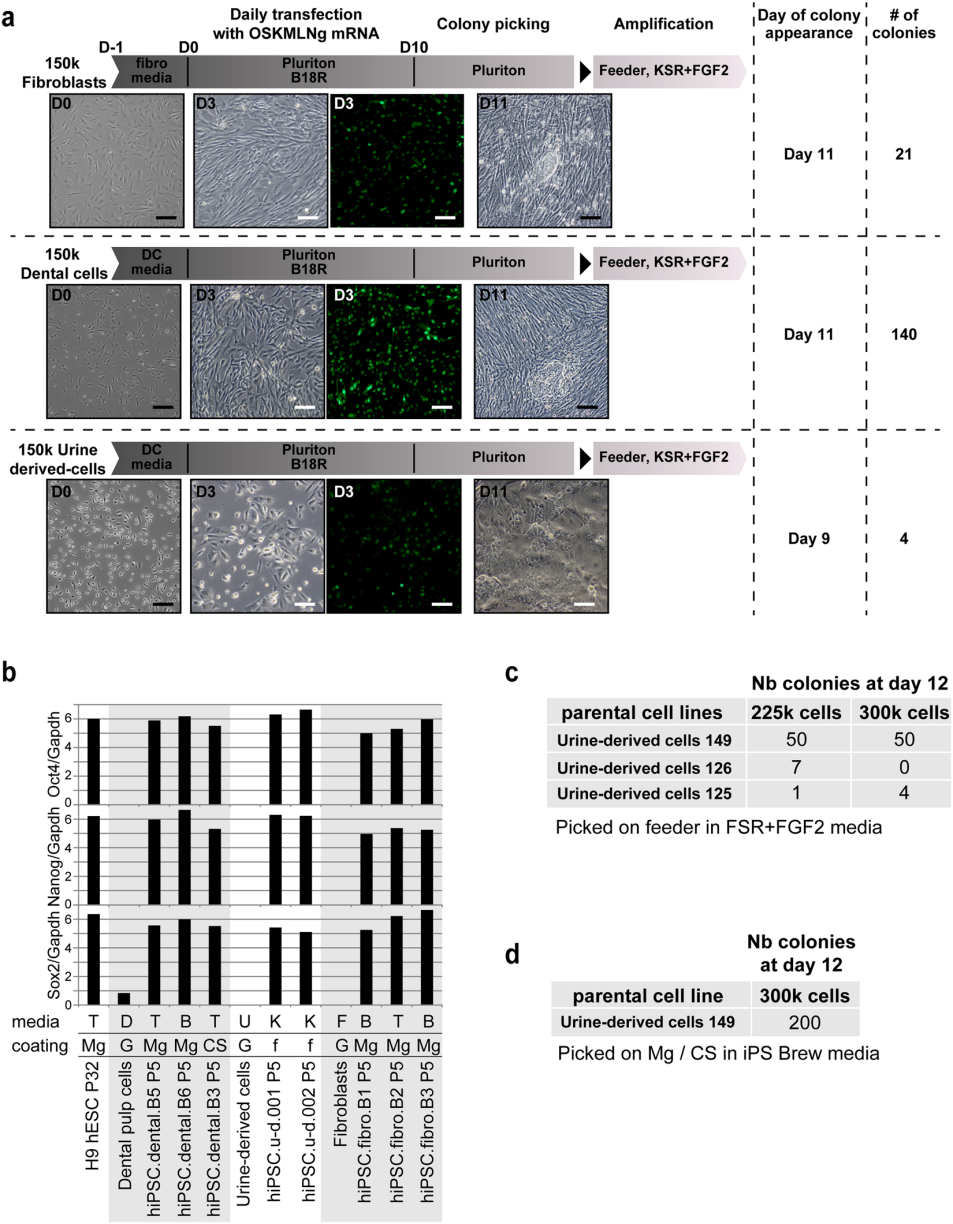
Optimization of mRNA reprogramming protocol for urine-derived cells. (**a**) Schematic view of the reprogramming protocols. 150,000 cells were seeded at day -1, then daily transfected with 625 ng mRNA cocktail containing Oct4, Sox2, Klf4, Myc, Lin28, Nanog and nGFP (OSKMLNg). Colonies started to appear between day 9 and day 11, and were picked on feeders (urine-derived cells) or Matrigel (dental cells and fibroblasts). White scale bar =50 µm, black scale bar =100 µm. (**b**) qPCR measurement of Oct4, Nanog and Sox2 expression in indicated cell lines. Media: TeSR1 (T), iPS-Brew XF (B), KSR + FGF2 (K), Dental cells media (D), urine-derived cells media (U), fibroblast media (F). Coating: feeders (f), Matrigel (Mg), Cell start (CS), gelatin (G). (**c**,**d**) Results of the reprogramming experiments that yielded clones analyzed in this study.

We sought to apply mRNA reprogramming on alternative cell types, that are easily accessible, such as dental pulp cells or urine-derived cells, a cell type that was recently used for reprogramming^16^. We used an in-house mRNA reprogramming protocol optimized for skin fibroblasts. The protocol comprised seeding of 150 000 cells, followed by daily transfection of 625 ng of Oct4, Sox2, Klf4, Myc, Nanog, Lin28 and nuclear GFP (nGFP) (OSKMNLg) for 11 days. This protocol allowed us to obtain 21 colonies from fibroblasts, and also worked on dental and urine-derived cells, yielding 140 and 4 colonies, respectively. We noticed that the transfection efficiency was high in all three cell types, as represented by GFP expression (Fig. 1a), despite the nGFP mRNA accounting for only 5% of the cocktail. Of note, urine-derived cells survived poorly at a starting cell density of 150,000 cells, in these transfection conditions, but hiPSC from those cells were the first to emerge (day 9 vs day 11 for skin fibroblasts and dental cells). Colonies from skin fibroblasts or dental cells were picked and transferred to feeder free conditions, in a variety of media and coating matrices, while urine-derived hiPSC colonies were transferred only to feeder cultures, due to the limited number of clones obtained (Fig. 1a). qPCR analysis of core pluripotency regulators Oct4, Sox2 and Nanog showed comparable expression levels to H9 hESC (Fig. 1b) at passage 5. For all cell sources, as no transgenes were present after day 11 in this reprogramming protocol, hiPSCs lines were readily established and could be banked at early passages. This allows to carry lines for a shorter time before validating them, therefore alleviating one of the hurdles in reprogramming which is extended passaging/sub cloning until no traces of transgenes can be detected.

We decided to focus on urine-derived cells as they can be obtained through one of the least invasive means and also because iPSCs derived from urine-derived cells have already been used to model multiple diseases^17–19^. To address the low survival rate and validate the reproducibility of our protocol, we increased the amount of cells seeded from 150,000 to 225,000 or 300,000 and also tested three additional patient cell lines (125, 126, 149). All test conditions yielded hiPSC colonies, except urine-derived cell line 126, which did not produce any colonies at a high starting cell density of 300,000 cells (Fig. 1c). Urine-derived cells are a heterogeneous population that varies a lot between cell preps and patients^20^. Thus, the derivation of iPSCs clones from multiple urine-derived cell lines demonstrates the robustness of the protocol. Finally, we repeated the reprogramming of patient 149 cells to establish hiPSC directly on Matrigel, a commonly used coating matrix. We were able to generate feeder-free hiPSC lines, as recently published for episomal reprogramming of urine-derived cells^21^ (Fig. 1d).

Altogether, our results showed that mRNA reprogramming can be used for multiple readily accessible cell types, including urine-derived cells, and that it is reproducible on 4 different patient cell lines.

### Validation of hiPSC established from bulk cultures

**Figure 2:**
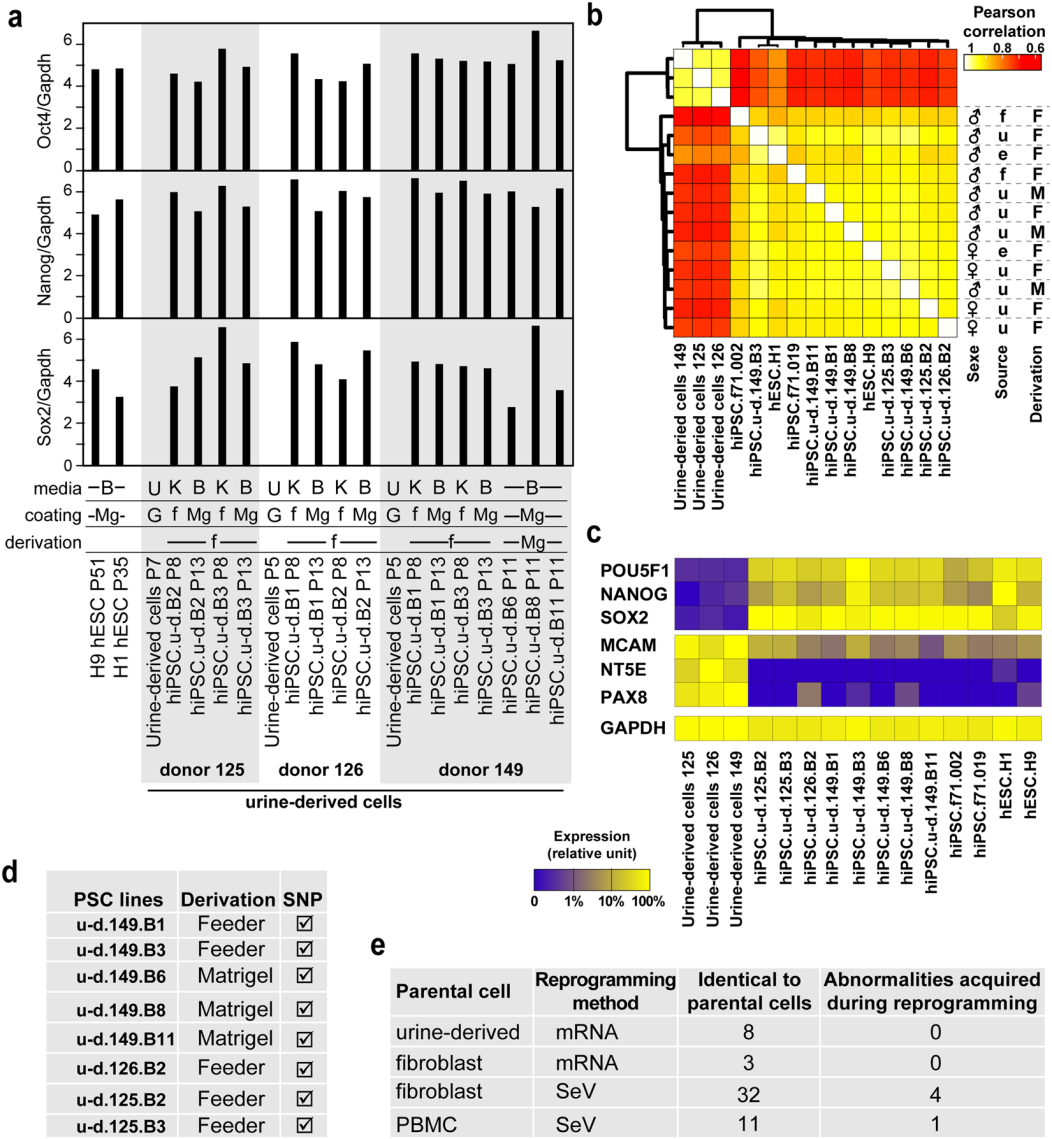
Characterization of feeder and feeder-free bulk hiPSC lines. (**a**) qPCR measurement of Oct4, Nanog and Sox2 expression in indicated cell lines. (**b**) Indicated cells were analyzed by DGE RNAseq and expression profiles were compared using Pearson correlation. (**c**) Expression profile for pluripotency (POU5F1, NANOG, SOX2), urine-derived cells (MCAM, NT5E, PAX8) or the housekeeping gene GAPDH are presented as a heatmap. (**d**) Summary of the SNP profile of indicated cells lines. A tick mark means identical to parental cells. (**e**) Summary of the SNP analysis of the hiPSC lines generated at the iPSC core facility of Nantes.

Another important question in the field of hiPSC is whether we should use clones originating from a single cell, or a bulk cell population originating from multiple parental cells. As recently demonstrated, the main variability factor lies within the inter-patient variability rather than the reprogramming method or the source of somatic cells^22–24^. Conventional reprogramming protocols involve picking, isolating, and culturing multiple colonies, a process that is highly time consuming. This procedure is followed primarily to select for clones without transgene insertions and genomic abnormalities. In this regard, mRNA reprogramming of urine-derived cells would be advantageous. Since mRNA reprogramming does not involve transgene insertions and has also been shown to result in relatively fewer genomic abnormalities^9^ in the resulting iPS cells, the number of colonies that need to be picked and screened will be significantly lower. With this reasoning, we decided to evaluate iPSCs derived from bulk reprogramming cells (hereafter, referred to as hiPSC bulks). Gene expression analysis by qPCR of core pluripotency regulators Oct4, Sox2 and Nanog showed expression levels comparable to those in H9 hESC (Fig. 2a), independent of the culture medium. There were also no apparent difference in expression levels between cells derived on feeders and subsequently cultured in defined media and cell directly derived in feeder-free conditions. To further assess the urine-derived hiPSC (u-d hiPSC), we analyzed them by digital gene expression (DGE) RNAseq^25,26^ and compared them with hESC lines H1 and H9, 2 clones from a fibroblast-derived hiPSCs, f71.002 and f71.019, and parental urine-derived cells. Unsupervised Pearson correlation analysis showed that u-d hiPSC were interspersed with hESC and the f71 hiPSCs (Fig. 2b). Moreover, the u-d hiPSC did not correlate more with the parental urine-derived cells than hESCs or the f71 hiPSCs. Finally, urine-derived cells specific genes MCAM, PAX8 and NT5E^21,27^ did not show persistence in u-d hiPSCs compared to hESCs or f71 hiPSCs (Fig. 2c).

Additionally, we assessed the copy number variation by analyzing our hiPSC lines by SNP chips. None of the hiPSC lines we tested gained copy number abnormalities during reprogramming (Fig. 2d). SNP analysis (molecular karyotypes) is used as a benchmark in core facilities (Stem Cell COREdinate session, ISSCR 2016). In comparison to the state-of-the-art feeder derivation, direct derivation in feeder-free conditions did not result in any additional abnormalities (Fig. 2d). Given that the sensitivity of the assay used for SNP analysis is around 15%^28^, we concluded that the vast majority of cells had a normal SNP profile. Therefore, in addition to clonal iPSC derivation, mRNA reprogramming would support derivation of bulk hiPSC lines, which has been proposed to be beneficial for the quality of hiPSC lines^29^. The bulk reprogramming method also significantly reduces manual labor in terms of time spent on cell culture.

Comparison of these results with those from previous reprogramming experiments of fibroblasts and PBMCs carried out at the iPSC core facility of Nantes, revealed that the mRNA reprogramming introduced fewer genomic abnormalities. In our previous reprogramming experiments, the average error rate with Sendai virus reprogramming is 10% (Fig. 2e). Those results were in line with previously published evaluation of Sendai and mRNA reprogramming^9^. This advantage of mRNA reprogramming in reducing genomic abnormalities in resulting iPSCs is of particular importance as recent reports showed that genomic instability of hiPSC lines is one of the main hurdles in translating iPSC cells to clinical applications^30^.

**Figure 3:**
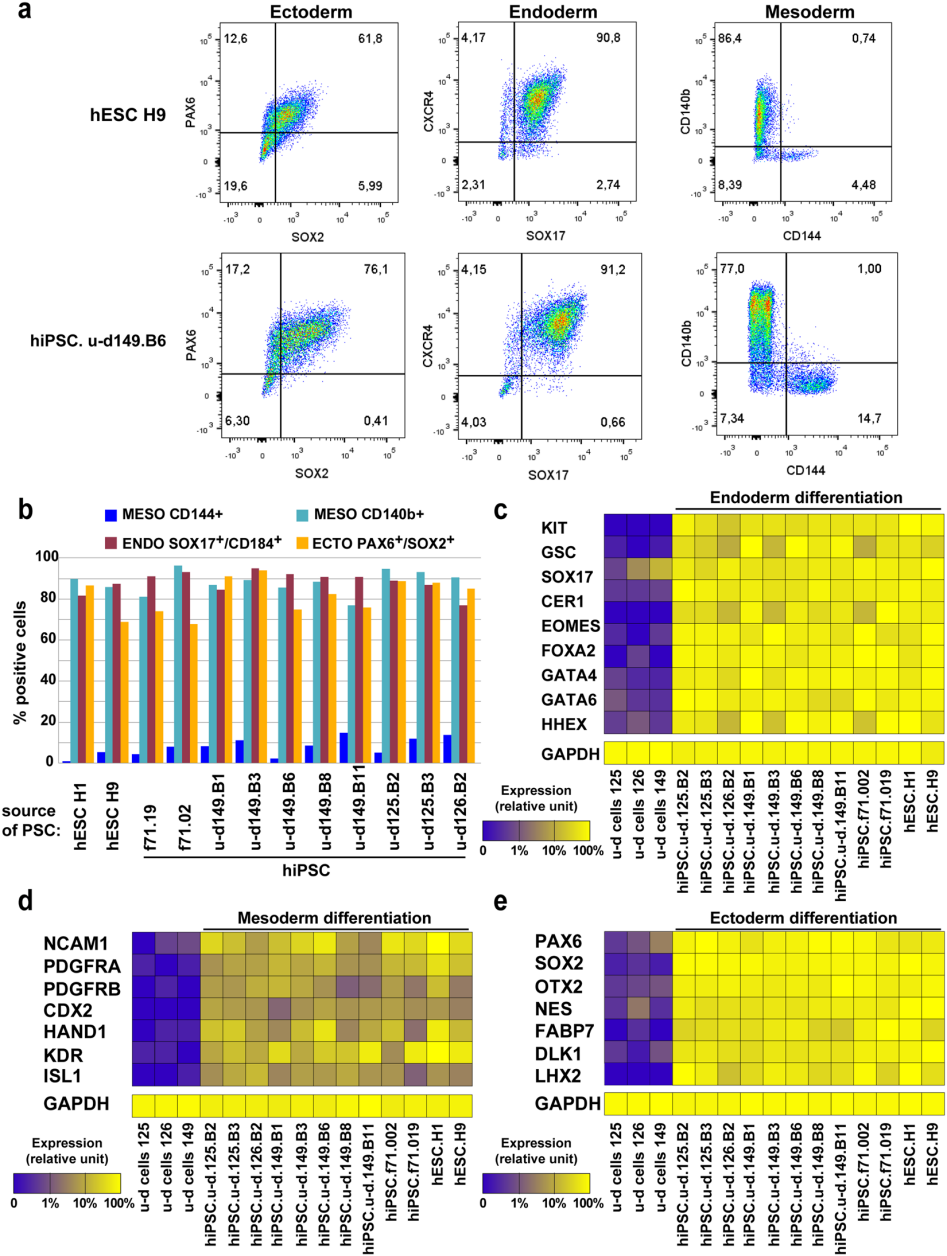
Early differentiation of feeder and feeder-free bulk hiPSC lines. (**a**) Flow cytometry profile of hESC H9 and hiPSC.u-d.149.B6 for ectoderm (PAX6/SOX2), endoderm (SOX17/CXCR4) and mesoderm (CD140b or CD144) germ layer after trilineage differentiation. (**b**) Summary of flow cytometry results obtained for 8 hiPSC.u-d, 2 hESC and 2 hiPSC.f71 lines. (**c**–**e**) Differentiated samples, as in (**a**), were analyzed by DGE RNAseq and selected gene expression representative of each germ layer were plotted as a heatmap, for endoderm (**c**), mesoderm (**d**) or ectoderm (**e**) genes.

To assess pluripotency of the hiPSC bulks that we generated, we performed differentiation into early germ layer intermediates. Cells were seeded as monolayers and induced in specific mesoderm, ectoderm and endoderm induction media for 7 days, and analyzed by flow cytometry for CD140 or CD144 (mesoderm), SOX2/PAX6 (ectoderm) and SOX17/CXCR4 (endoderm). hESC of u-d hiPSC line 149.B11 show typical staining with around 80% CD140b+, 10% CD144+, 75% SOX2+/PAX6+ and 85% SOX17+/CXCR4+ cells (Fig. 3a). The other tested hPSC lines showed similar results, demonstrating no adverse effect of the source of cells on the efficacy of engagement into the 3 germ layers (Fig. 3b). Finally, we investigated the expression levels of lineage specific genes by DGE RNAseq in differentiated cells from all PSC lines, and showed comparable expression levels for all PSC lines, supporting again that the u-d hiPSCs lines did not have differentiation biases for endoderm, mesoderm or ectoderm lineages (Fig. 3c–e).

**Figure 4:**
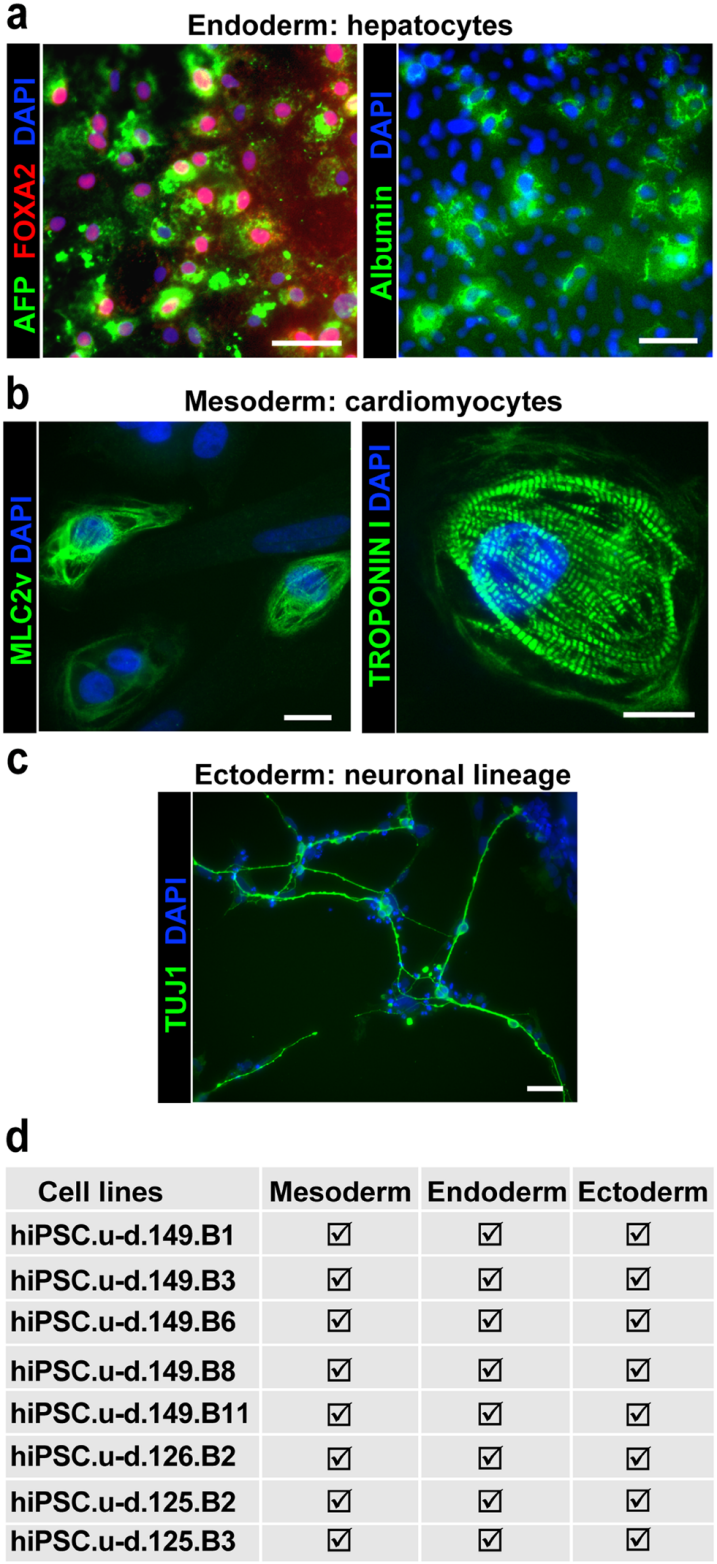
Advanced differentiation of feeder and feeder-free bulk hiPSC lines. (**a**) Hepatocytes differentiated from hiPSC (149.B11 and 149.B6) were stained for AFP and FOXA2 (left) or Albumin and HNF4 (right). Scale bar = 50 µm. (**b**) Cardiomyocytes differentiated from hiPSC (149.B6) were stained for MLC2V (left) and Troponin I (right). Scale bar = 16 µm. (**c**) Neurons differentiated from hiPSC (149.B11) were stained for TUJ1. Scale bar = 50 µm. (**d**) Summary table of all differentiation achieved on indicated cell lines.

To further show the differentiation potential of our cells, we performed directed differentiation into hepatocytes, cardiomyocytes and cells of neuronal lineage. The hepatocyte differentiation protocol has been previously described^31^. The staining showed large number of cells expressing FOXA2, alpha-foeto protein (AFP) and Albumin (Fig. 4a), typical for hepatocytes differentiated from hiPSCs. Cardiomyocytes were differentiated according to our protocol^17^, and monitored for beating colonies, and characterized at day 28 by immunofluorescence for MLC2v, Troponin I (Fig. 4b). Staining showed characteristic striated structures. Finally, we assessed ectoderm germ layer differentiation by differentiating hiPSC into neurons and stained for Tuj1 (Fig. 4c). The staining revealed axon-like patterns, typical for neurons. Each of the 8 hiPSCs bulks, originating form 3 different patient lines, were subjected to these differentiation protocols and showed proper differentiation (Fig. 4d).

## Discussion

Our results show that mRNA reprogramming allows the derivation of iPSCs from urine-derived cells, in as little as 9 days. Moreover, urine-collection is the least invasive way to obtain biological samples amenable for reprogramming. The resulting iPSCs did not present any genomic abnormalities, reinforcing that shorter duration of reprogramming introduces fewer genomic abnormalities in cells^2^. Readily available cell source and less labor-intensive protocols are key for the wide-spread use of hiPSCs, with more and more patient lines in studies. Our protocol addresses both of these factors. We showed that mRNA reprogramming is less labor-intensive by allowing feeder-free reprogramming^11–13^, supporting derivation of bulk iPSCs, and reducing the number of clones that need to be screened for genomic abnormalities. We have also presented evidence for the applicability of this reprogramming method on readily available cells sources such as urine-derived cells.

One of the expectation of hiPSCs is their use for clinical treatments. Completely defined, xeno-free, media are available for mRNA reprogramming, in combination with GMP compatible coating matrixes^12,13^. Multiple countries are setting up programs to generate HLA-typed cells banks. However, we have to be able to check that the selected donors have genomes compatible with high-quality differentiated cells for a broad spectrum of differentiation. To address the need of immunologically-matched hiPSCs, we envision to generate hiPSCs from urine-derived cells of donors with specific HLA haplotypes. In clinical settings, clone picking will be preferred. We could generate HLA specific hiPSCs, validate that they are producing high-quality differentiated cells, and repeat the reprogramming of specific donors under GMP grade conditions. Indeed, this strategy is in line with a recent analysis showing that the major parameter influencing the variability between PSC lines is the genomic background of the donor^32^. When realized, this will be a time- and labor-effective method to derive hiPSCs for use in clinical trials. Moreover, it allows for generating immune-defined hiPSCs at lower costs, thus enabling their use in preclinical studies aimed at investigating immune-tolerance of hiPSCs progeny, a question that requires thorough investigation^2^.

Other protocols improving mRNA reprogramming have recently emerged^33^. Yoshioka and colleagues used semi-replicative polycystronic mRNA (srRNA) harboring reprogramming factors to reprogram somatic cells. The srRNA allows reprogramming of CD34+ circulating cells. However, it suffers from two caveats that we have sought to overcome with our method: (1) it requires significant quantity of blood to obtain sufficient adherent cells to reprogram, (2) the transgenes are present until passage 6, which nullifies one of the main advantages of mRNA reprogramming in our view. It will be interesting to follow the improvements upon this method, particularly those aimed at reducing the number of days that transgenes are present in reprogramming.

HiPSCs have become a useful complement to hESCs in studying human development and physiopathology and in developing new regenerative medicine treatments. The reprogramming process generates iPSC clones of distinct immunogenic, genetic, epigenetic, and functional qualities from which good clones need to be selected prior to using these cells in molecular and biomedical applications. However, screening for good clones can become an arduous and expensive task that might be ameliorated by enhancing the reprogramming fidelity of the generated iPSCs. Improvement of reprogramming techniques, in particular their kinetics and efficiency, will have a direct effect on the quality of reprograming since they reduce the work needed to screen and select good clones for downstream applications. Thus, we propose mRNA reprogramming of urine-derived cells as a valuable resource for the scientific community to accelerate the development of hiPSCs-based regenerative medicine protocols.

## Material and Methods

### Tissue culture

Urine samples were collected in a 250 ml bottle previously conditioned with 10% of RE/MC medium (see below) for storage (up to 24 h at 4 °C) and transported as previously described^34^. RE/MC (1:1) medium was prepared by mixing RE medium (Renal epithelial cell growth medium SingleQuot kit supplement and growth factors; Lonza) with MC (mesenchymal cell) medium prepared separately. MC medium is composed of DMEM/high glucose medium (Hyclone) supplemented with 10% (vol/vol) FBS (Hyclone), 1% (vol/vol) GlutaMAX (Life Technologies), 1% (vol/vol) NEAA (Life Technologies), 100 U/ml penicillin (Life Technologies), 100 μg/ml streptomycin (Life Technologies), 5 ng/ml FGF2 (Miltenyi Biotec), 5 ng/ml PDGF-AB (Cell Guidance Systems) and 5 ng/ml EGF (Peprotech). U-cells were isolated from urine samples and cultured accordingly to the procedure described in^16^ with slight modifications. Briefly, urine samples were centrifuged 5 min at 1200 g and the pellet was washed with pre-warmed DPBS (Gibco) containing 100 U/ml penicillin and 100 μg/ml streptomycin (Gibco). Pellets were resuspended in 2 ml RE/MC proliferation medium and cultured on 0.1% gelatin-coated six-well plates. Cells were incubated at 37 °C in normoxia (20% O2, 5%CO2) for 4–5 days without any change of medium nor moving. Urine-derived cells were further passaged using TrypLE Express (Gibco) and expanded in RE/MC medium with daily change of half of the media.

Dental cells were kindly provided by Clinident. Dental pulp cells were cultured in high glucose alpha MEM, 2 mM L-Glutamin, 20% fetal bovine serum, 100 µM ascorbic acid, 1% pen/strep (Life Technologies), and passed with trypsin/EDTA (Life Technologies).

Fibroblasts were obtained from Lonza (Cat# CC-2511 Lot 0000293971). Fibroblasts were cultured in high glucose DMEM, Glutamax II, 10% fetal bovine serum, 1% sodium pyruvate and 1% non-essential amino acids).

Pluripotent stem cells on feeder cells were cultured in KSR medium (DMEM/F-12, 20% Knockout^TM^ serum replacement, 1% non-essential amino acids, 1% glutamax, 50 µM 2-mercaptoethanol and 10 ng/ml Fibroblast growth factor 2 (Peprotech)). They were mechanically passaged by cutting colonies with a needle. hESC lines WA01 or H1 (Lot WB0111) and WA09 or H9 (Lot WB0090) were obtained from WiCell, under authorization RE13-004 from the Agence de la Biomédecine. Pluripotent stem cells in feeder-free conditions were cultured on Matrigel (BD/Corning) in mTeSR1 media (Stem Cell Technologies) or StemMACS^TM^ iPS-Brew XF (Miltenyi Biotec); cells were non-enzymatically dissociated with StemMACS Passaging Solution XF (Miltenyi Biotec).

All cells were cultured at 37 °C under 20%O2 and 5%CO2.

### Reprogramming

Cells are seeded on Matrigel-coated wells on day −1 in their usual culture media, and are daily transfected from day 0 to day 10, with 625 ng of mRNA cocktail (38% Oct4, 11.4% Sox2, 12.7% Klf4, 10.1% Lin28, 12.7% Myc, 10.1% Nanog, 5.1% nGFP, Milenyi Biotec) in Pluriton media (Reprocell) supplemented with 4 ng/ml FGF2 (Peprotech) and 200 ng/ml B18R (eBioscience). From day 11, cells are cultured in Pluriton supplemented with 4 ng/ml FGF2. Colonies were picked on feeders in KSR + FGF2 media or directly on Matrigel-coated dishes in TeSR1 or iPS Brew. All colonies that are picked at a given time point are pooled together, and termed “bulk”, with a name finishing by “B” and a 2 digit number.

### Early germ layer differentiation

hPSC lines were differentiated into endoderm, mesoderm and ectoderm using Stemmacs Trilineage Kit (Miltenyi biotec). 80,000 cells for mesoderm, 130,000 cells for endoderm and 100,000 cells for ectoderm were plated in 24 wells plates, and cultured in specific media for 7 days, as specified by the protocol. On day 7, differentiated cells were analysed by flow cytometry and DGE RNAseq.

### Advanced Differentiation

#### Hepatocytes

We followed the differentiation protocol published in^19^. In brief, were plated in tissue-culture plates previously coated with Matrigel at 0.05 mg/ml for 1 h, and cultured in mTSeR or StemMACS^TM^ iPS-Brew XF. Once cells reached 70–80% confluence, differentiation was performed with RPMI 1640 (Life Technologies) and B27 (Life Technologies) containing Activin A (AA; Miltenyi Biotec), FGF2, BMP4 (Miltenyi Biotec) and HGF (Miltenyi Biotec) with the following sequence: AA/BMP4/FGF2 (2 days); AA (3 days), BMP4/FGF2 (5 days) and HGF (5 days). Cells were then incubated with Hepatocyte Culture Medium (Lonza) supplemented with oncostatin M (Miltenyi Biotec) for 5 days.

#### Cardiomyocytes

Human iPS cells were differentiated into CMs using the established matrix sandwich method^35^ with modifications. Briefly, 6 days before initiating differentiation, hiPS cell colonies were passaged on human embryonic stem cell–qualified Matrigel-coated plates (0.05 mg/mL; BD Biosciences) using Gentle Cell Dissociation Buffer (Stemcell Technologies) and cultured as a monolayer in mTeSR1 with 1× Y-27632 ROCK inhibitor (Stemcell Technologies) in a normal oxygen atmosphere. When cells reached 80% confluence, cold mTeSR1 with Growth Factor Reduced Matrigel (0.033 mg/mL) was added to create an overlay of Matrigel. Differentiation was initiated 24 hours later (day 0) by culturing the cells in RPMI-1640 medium (Life Technologies) supplemented with B27 (without insulin; Life Technologies), 100 ng/mL activin A (Miltenyi Biotec), and 10 ng/mL FGF2 (Miltenyi Biotec) for 24 hours. On the next day, the medium was replaced by RPMI-1640 medium supplemented with B27 without insulin, 10 ng/mL BMP4 (Miltenyi Biotec), and 5 ng/mL FGF2 for 4 days. By day 5, cells were cultured in RPMI-1640 medium supplemented with B27 complete (Life Technologies) and 1% NEAA and changed every 2 to 3 days.

#### Neuron lineage

Cells were scrapped to form embryoid bodies in APEL media (Stem Cell Technologies). At day 5, cells were transferred on regular tissue culture dishes and cultured in N2B27 supplemented with 1 µM Retinoic Acid. At day 12, cells were fixed and analyzed by immunofluorescence.

### Immunofluorescence

After formaldehyde 4% fixation, cells were permeabilized with 0.5% saponin, blocked with 1% PBS-BSA and stained with primary antibodies at 4 °C, overnight. Secondary antibody staining was performed at room temperature, for 1 hour, in parallel with DAPI counter-staining. Cells were imaged on a Leica epifluorescence microscope.

#### Primary antibodies

Troponin I (Santa Cruz Biotechnology), MLC2v (Proteintech Europe), Tuj1 (Santa-Cruz), FOXA2 (Santa Cruz), Alpha foeto protein (Sigma-Aldrich), Albumin (Cedarlane).

#### Secondary antibodies

Alexa 488– and Alexa 568–conjugated antibodies (Molecular Probes).

### Flow cytometry

Cells from differentiation pathways were dissociated using 0.05%Trypsin/EDTA (Gibco). Cells were blocked using 5% Bovine Serum Albumin (BSA; Sigma-Aldrich) in PBS (Gibco). Cells were stained with a CD144 VE-cadherin-FITC (1:11, 130-100-713 Miltenyi) and CD140b-APC (1:11, 130-105-322 Miltenyi) for mesodermal lineage. Cells were stained with a CD184 CXCR4-APC (1:11, 130-109-886 Miltenyi) and Anti-Sox17-Vio (1:50, 130-111-147 Miltenyi) for endodermal lineage. Cells were stained with an Anti-PAX-6-APC (1:11, 130-107-829 Miltenyi) and Anti-Sox2-FITC (1:11, 130-104-993 Miltenyi) for ectodermal lineage. Cells were analyzed using a LSR™.

### RT-qPCR analysis

Total RNA was extracted using RNeasy® columns and DNAse-treated using RNase-free DNase (Qiagen). For quantitative PCR, first-strand cDNAs were generated using 500 ng of RNA, M-MLV reverse transcriptase (Invitrogen), 25 µg/ml polydT and 9.6 µg/ml random primers (Invitrogen).

To quantitate transcripts, absolute quantitative PCR was performed on a Viia 7 (Applied Biosystems) using power SYBR green PCR master mix (Applied Biosystems), for genes listed in Table 1. For each sample, the ratio of specific mRNA level relative to GAPDH levels was calculated. Experimental results are shown as levels of mRNA relative to the highest value.

**Table 1:** PCR PRIMERS.

Gene Name	Primer sequence 5′-3′	Amplicon size (bp)	Melting Temp. (°C)	Position
GAPDH	AATCCCATCACCATCTTCCA TGGACTCCACGACGTACTCA	82	80.5	494–576
OCT4	TGGGTGGAGGAAGCTGACAACAAT TTCGGGCACTGCAGGAACAAATTC	142	82.1	1005–1147
SOX2	CCTACTCGCAGCAGGGCACC CTCGGCGCCGGGGAGATACA	169	xx	1114–1283
NANOG	ATAGCAATGGTGTGACGCAGAAGG CTGGTTGCTCCACATTGGAAGGTT	116	82	701–816

All primers have a hybridization temperature of 60 °C. GAPDH, OCT4 and NANOG amplicons span two adjacent exons.

### Expression profiling by DGE-seq

3′digital gene expression (3′DGE) RNA-sequencing protocol was performed according to^25^. Briefly, the libraries were prepared from 10 ng of total RNA. The mRNA poly(A) tail were tagged with universal adapters, well-specific barcodes and unique molecular identifiers (UMIs) during template-switching reverse transcriptase. Barcoded cDNAs from multiple samples were then pooled, amplified and tagmented using a transposon-fragmentation approach which enriches for 3′ends of cDNA. A library of 350–800 bp was run on an Illumina HiSeq. 2500 using a Hiseq Rapid SBS Kit v2–50 cycles (ref FC-402–4022) and a Hiseq Rapid PE Cluster Kit v2 (ref PE-402–4002).

Read pairs used for analysis matched the following criteria: all sixteen bases of the first read had quality scores of at least 10 and the first six bases correspond exactly to a designed well-specific barcode. The second reads were aligned to RefSeq human mRNA sequences (hg19) using bwa version 0.7.4 4 with non-default parameter “-l 24”. Reads mapping to several positions into the genome were filtered out from the analysis. Digital gene expression (DGE) profiles were generated by counting for each sample, the number of unique UMIs associated with each RefSeq genes. DGE-sequenced samples were acquired from three sequencing runs. All sequenced samples were retained for further analysis.

DESeq 2 was used to normalize expression with the DESeq function. Normalized counts were transformed with vst (variance stabilized transformation) function from DESeq library. This log-like transformation was used for variance analysis.

### SNP analysis

DNA was extracted from somatic and iPSCs samples using the QIAGEN QiaAmp kit, according to the manufacturer’s recommendations. The gDNA was quantified and qualified using a nanodrop. 200 ng of gDNA was outsourced to Integragen Company (Evry, France) for karyotype analysis using HumanCore-24-v1 SNP arrays. This array contains over 300000 probes distributed throughout the genome with a median coverage of one probe every bases 5700 bases. All genomic positions were based upon Human Genome Build 37 (hg19).

DNA samples were hybridized on HumanCore-24-V1 SNP arrays according to the manufacturer’s instructions by Integragen. Analysis was performed with GenomeStudio software. Chromosome abnormalities were determined by visual inspection of logR ratios and B-allele frequencies (BAF) values and comparing parental cells and iPS-derived samples. LogR ratio, the ratio between observed and expected probe intensity, is informative regarding copy number variation (i.e. deletions/duplications) and BAF is informative regarding heterozygosity. We used the SNP data to compute CNV. In particular, this type of chips allows to detect loss of heterozigocy (LOH), an important concern for hiPSC, which is not possible with classical CGH arrays.

### Ethical approval and informed consent

All experiments were carried out in accordance with french guidelines and regulations. All patients gave informed consents. Reprogramming of patient samples was approved by the French ministry of higher education and research, under No. DC-2011-1399. hESC H1 and H9 were used under agreement FE13-004 from Agence de la Biomédecine.

## Data Availability

The raw read sequence data and sample annotations generated for this paper are available at European Nucleotide Archive (ENA) with accession number (under upload).
